# Mild simulator sickness can alter heart rate variability, mental workload, and learning outcomes in a 360° virtual reality application for medical education: a post hoc analysis of a randomized controlled trial

**DOI:** 10.1007/s10055-022-00688-6

**Published:** 2022-09-14

**Authors:** Li-Jen Hsin, Yi-Ping Chao, Hai-Hua Chuang, Terry B. J. Kuo, Cheryl C. H. Yang, Chung-Guei Huang, Chung-Jan Kang, Wan-Ni Lin, Tuan-Jen Fang, Hsueh-Yu Li, Li-Ang Lee

**Affiliations:** 1 Department of Otorhinolaryngology-Head and Neck Surgery, Sleep Center, Linkou Medical Center, Linkou Chang Gung Memorial Hospital, No. 5, Fu-Hsing Street, Gueishan District, Taoyuan City, 33305 Taiwan, Republic of China; 2 Faculty of Medicine, Graduate Institute of Clinical Medicine Sciences, Chang Gung University, Taoyuan, 33302 Taiwan; 3 Department of Computer Science and Information Engineering, Graduate Institute of Biomedical Engineering, Chang Gung University, Taoyuan, 33302 Taiwan; 4 Department of Neurology, Linkou Chang Gung Memorial Hospital, Taoyuan, 33305 Taiwan; 5 Department of Family Medicine, Taipei Branch and Linkou Main Branch, Linkou Medical Center, Chang Gung Memorial Hospital, No. 5, Fu-Hsing Street, Gueishan District, Taoyuan, 33305 Taiwan, Republic of China; 6 Department of Industrial Engineering and Management, National Taipei University of Technology, Taipei, 10608 Taiwan; 7 Institute of Brain Science, National Yang Ming Chiao Tung University, Taipei, 11221 Taiwan; 8 Department of Laboratory Medicine, Linkou Chang Gung Memorial Hospital, Taoyuan, 33305 Taiwan; 9 Department of Medical Biotechnology and Laboratory Science, Graduate Institute of Biomedical Sciences, Chang Gung University, Taoyuan, 33302 Taiwan; 10 School of Medicine, National Tsing Hua University, Hsinchu, 300044 Taiwan

**Keywords:** Heart rate variability, Mini-clinical evaluation exercise, Simulator sickness, Task load index, Visual reality, 360° video

## Abstract

Virtual reality (VR) applications could be beneficial for education, training, and treatment. However, VR may induce symptoms of simulator sickness (SS) such as difficulty focusing, difficulty concentrating, or dizziness that could impair autonomic nervous system function, affect mental workload, and worsen interventional outcomes. In the original randomized controlled trial, which explored the effectiveness of using a 360° VR video versus a two-dimensional VR video to learn history taking and physical examination skills, only the former group participants had SS. Therefore, 28 undergraduate medical students who participated in a 360° VR learning module were included in this post hoc study using a repeated measures design. Data of the Simulator Sickness Questionnaire (SSQ), heart rate variability (HRV) analysis, Task Load Index, and Mini-Clinical Evaluation Exercise were retrospectively reviewed and statistically analyzed. Ten (36%) participants had mild SS (total score > 0 and ≤ 20), and 18 (64%) had no SS symptom. Total SSQ score was positively related to the very low frequency (VLF) band power, physical demand subscale, and frustration subscale, and inversely related to physical examination score. Using multilevel modeling, the VLF power mediated the relationship between total SSQ score and physical examination score. Furthermore, frustration subscale moderated the mediating effects of the VLF power. Our results highlight the importance of documenting SS to evaluate a 360° VR training program. Furthermore, the combination of HRV analysis with mental workload measurement and outcome assessments provided the important clinical value in evaluating the effects of SS in VR applications in medical education.

## Introduction

### Virtual reality and its potential in medical education

Virtual reality (VR) technologies have been used to serve different purposes in a wide range of industries, including healthcare. One of its potential applications lies in medical education since VR can accurately simulate an externally valid, safe, reversible, and repeatable environment, which is not possible to be created otherwise in the real world (H. S. Lee et al 2020). This not only allows both the learners and educators to be more engaged in the training program, but also has a particular contribution to infection control for contagious diseases like COVID-19.

Currently, general learners can use VR to improve learning outcomes (Dyer et al 2018; Frendø et al 2020; Wu et al 2020). Furthermore, the evaluation of VR applications in higher education, such as medical education, has shifted from usability to learning outcomes (Radianti et al 2020; Chen et al 2020; Howard et al. 2020; Kaplan et al 2021). Moreover, among those studies which utilized VR or augmented reality in medical education, the majority (48–89%) was conducted for training on invasive procedures or knowledge highly related to spatial sense, such as anatomy, radiological image, laparoscopic surgery, and other operations (Samadbeik et al 2018; Ammanuel et al 2019; Tang et al 2020). For history taking and physical examination (H&P), which is traditionally considered not possible to be achieved without person-to-person interactions, VR is still a relatively new application. Yet, H&P are fundamental skills for healthcare professionals in almost all fields, and the implementation of H&P training may be refined to catch up with the pace of changes in clinical practice (J. Pottle 2019), especially in light of the COVID-19 pandemic.

### Simulator sickness as an obstacle to the application of virtual reality

One of the biggest barriers to widely harnessing VR is unpleasant user experiences. Simulator sickness (SS) is a syndrome sometimes users develop during VR exposure, especially with immersive VR. While non-immersive VR let users experience or interact with a computer-created environment without feeling immersed in it, immersive VR is characterized by a 3D realistic simulation which enables the user to feel immersed in this artificial world, fully engage with the generated environment, and be isolated from the actual surroundings (Ventura et al 2019; O'Sullivan et al 2018). SS has been reported to be a limitation of immersive VR to improve simulation outcomes (Menin et al 2018).

The main symptoms of SS include general discomfort, tiredness, headache, fullness in the head, difficulty concentrating, disorientation, eye fatigue, blurred vision, drooling, sweating, nausea, dizziness, vertigo, awareness of the stomach, and belching (Duzmanska et al 2018; Kennedy et al 1993; H. G. Kim et al 2019). SS is one of the main themes in negative public reviews of VR systems (Faric et al 2019), and up to 35% of VR users have been reported to experience SS, as assessed by subjective questionnaires in various VR environments (Treleaven et al 2015; Tychsen and Foeller 2020; Ungs 1989). A younger age, female sex, stress, anxiety, lack of SS, and a wider field of view of the simulator have been reported to increase the occurrence and severity of SS (Kolasinski 1995).

In a pilot study of medical students learning H&P via a head-mounted display (HMD), no one (0/13) had SS among those who watched a 2D video, while some (3/11) experienced mild SS and had worse learning outcomes among those who watched a 360° VR video (L. A. Lee et al 2018). Furthermore, SS generates unequal effects across multiple individual differences, and these effects arise resilient across VR applications and HMD technologies (Saredakis et al 2020; Howard et al. 2021). Notably, resolving users’ uncomfortable experiences is a key issue in improving VR learning outcomes (Duzmanska et al 2018; H. G. Kim et al 2019).

Various subjective measures, such as the Simulator Sickness Questionnaire (SSQ) (Kennedy et al 1993) and Virtual Reality Sickness Questionnaire (H. K. Kim et al 2018a, b), have been developed to assess SS. The SSQ has been demonstrated to be reliable and valid and is currently considered the gold standard assessment tool for SS symptoms (Keshavarz and Hecht 2011a). Some studies have also used physiological measures such as electroencephalogram rhythm energy ratio (Li et al 2019) and heart rate variability (HRV) (Ohyama et al 2007) to evaluate SS objectively. However, the association between objective physiological measures and subjective SS symptoms remains inconclusive (Duzmanska et al 2018).

### Heart rate variability as a possible measure for evaluating physiological responses to simulator sickness

The autonomic nervous system (ANS) is connected to our central nervous system and has widespread peripheral innervation to almost every organ system in the human body. It responds to internal and external stimuli, including what an individual sees, hears, smells, feels, and thinks. The ANS then reacts promptly through a highly intricate and complex network so that the individual can adapt to the changes from the environment or the body itself (C.H. Gibbons 2019). Activation of the ANS, mainly the sympathetic nervous system in terms of pupil dilation (Juvrud et al 2018) and decreased heart period (Min et al 2004), is frequently observed in immersive VR applications. However, the association between the ANS evaluations and SS severity remains inconclusive (Miller et al 1993; Min et al 2004).

HRV is a simple, noninvasive tool to evaluate the cardiac ANS; smaller HRV values are associated with suboptimal adaptation with ANS insufficiency, whereas larger HRV values are related to good adaptation with an efficient ANS mechanism (Alvares et al 2013; H. G. Kim et al 2018a, b; Kuo et al 1999). Some previous researches have reported links between SS and HRV. Ohyama et al. demonstrated that during VR immersion, young volunteers with SS had gradually worsening subjective symptoms and a gradual increase in low frequency (LF) power (Ohyama et al 2007); however, subjective symptoms were not correlated with LF power. In another pilot study, three participants with SS had a larger power of the very low frequency (VLF) band and frustration score than eight without SS after 360° VR training (L. A. Lee et al 2018). Real-time monitoring of HRV may potentially identify the onset and progression of SS during 360° VR training as an objective physiological parameter. Furthermore, HRV indices have been linked to learning outcomes in medical students (Yoo et al 2021).

### Mental workload as a subjective parameter to evaluate the impact of simulator sickness

Mental workload is one of the cognitive demands associated with novices’ performance in many learning tasks (Barre et al 2019; Dias et al 2018; Klein et al 2012). Although mental workload has not been clearly defined, it refers to interactions of mental demands imposed on learners by tasks they participate (Loft et al 2007). The increased mental workload increases the number of errors and decreases skill acquisition during surgical training (Lau et al 2020).

Subjective measures, primary tasks, secondary tasks, and physiological measures have been used to measure mental workload (Foo et al 2013). For example, the NASA Task Load Index (NASA TLX) is a validated mental workload assessment (Hart and Staveland 1988) that has been widely used in many studies (Hart 2006). VR could reduce mental workload as assessed using the NASA TLX questionnaire in surgical training (Barre et al 2019) and procedural errors in the operating room (Seymour et al 2002). However, immersive VR simulation training for laparoscopy might result in worse performance than conventional VR simulation training because of inducing a larger mental workload (Frederiksen et al. 2020). Nevertheless, the association between SS and mental workload during a VR training program has not been comprehensively investigated.

### Hypothesis and aims of the current study

We hypothesized that for participants who experience SS during a VR training program, the SS would: (1) be associated with HRV as an objective physiological measure, (2) be associated with mental workload as a subjective cognitive parameter, and (3) have negative impacts on learning outcomes of H&P skills both directly and indirectly through mediation of objective physiological and subjective cognitive changes.

The study aimed to investigate the effects of SS on autonomic function, mental workload, and learning outcomes by examining HRV, NASA TLX questionnaire, and the Mini-Clinical Evaluation Exercise (Mini-CEX) scoring sheet during and after a 360° VR H&P learning program among a sample of healthy undergraduate medical students.

## Methods

### Ethical considerations

This study was a retrospective case series analysis. A post hoc analysis was performed to statistically analyze the data of undergraduate medical students who had undergone a 360° VR learning program in the randomized controlled trial for exploring the effectiveness of using a 360° versus a two-dimensional (2D) video training program to learn history taking and physical examination skills from March 1, 2018, to December 31, 2019, at an academic teaching hospital (Chang Gung Memorial Hospital, Linkou Main Branch, Taoyuan, Taiwan) (Chao et al. 2022). The Institutional Review Board of Chang Gung Medical Foundation approved this study (No: 201601821B0), and all procedures were conducted in compliance with the Declaration of Helsinki 1975. The participants provided written informed consent.

### Research design

**Fig. 1:**
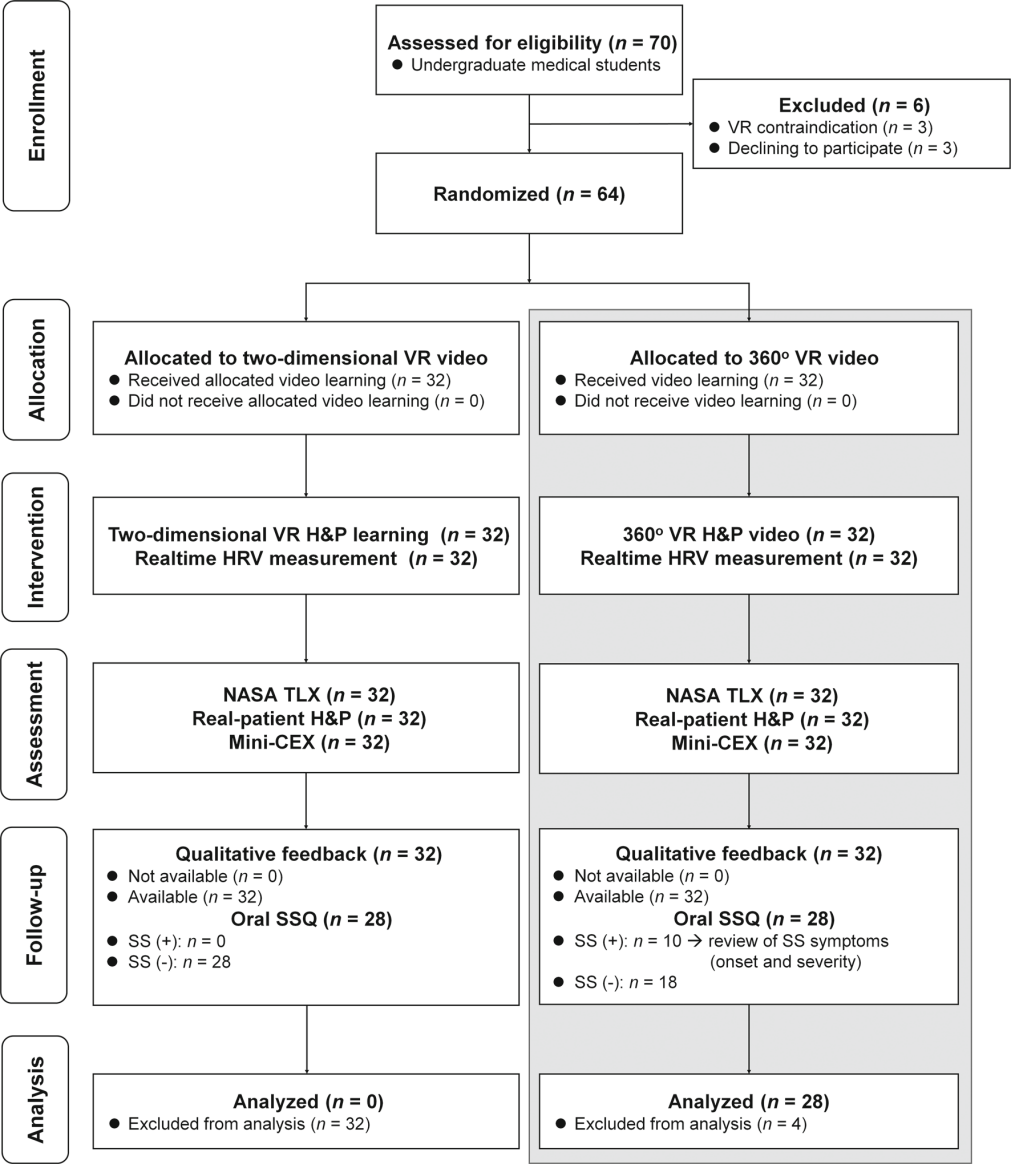
Study flowchart showing undergraduate medical students underwent 360° virtual reality (VR) history taking and physical examination (H&P) learning with or without simulator sickness (SS) (gray box). White boxes indicate the original randomized controlled trial (Chao et al. 2022). Abbreviations: HRV: heart rate variability; Mini-CEX: Mini-Clinical Evaluation Exercise; NASA TLX: NASA task load index; SSQ: Simulator Sickness Questionnaire

In the original study (Chao et al. 2022), 64 senior undergraduate medical students were randomized to receive a 10-min immersive 360° (360° VR video group; *n* = 32) or 2D VR instructional video (2D VR video group; *n* = 32), including essential knowledge and competency of H&P, using a between-subject design. The two groups were matched by age, sex, and cognitive style. In the first block of the original study (*n* = 8), one of the four 360° VR video participants reflected having mild SS while watching 360° VR video, whereas none of the four 2D VR video participants had SS throughout the intervention. After this index case, the research team used the oral SSQ to evaluate SS during qualitative feedback. Furthermore, only the 360° VR video participants experienced SS, and none of the 2D VR video participants had SS after the original study finished. Accordingly, this post hoc analysis included the 360° VR video participants who had completed the oral SSQ (*n* = 28) using a repeated measures design. Figure 1 demonstrates the study flowchart.

### Participants

Twenty-eight healthy undergraduate medical students who were novice learners of otorhinolaryngology-head and neck surgery (ORL-HNS) had been prospectively recruited in the original study. One of the authors held information meetings about the study (including 16 SS symptoms), and interested participants were subsequently recruited into the original study. The original inclusion criteria were: (1) age > 20 years, and (2) novices in ORL-HNS. The main exclusion criteria were: (1) recent SS, (2) heart conditions, or (3) epileptic symptoms.

### Virtual reality learning program

The principles of designing effective instruction (Morrison et al 2013) were followed to develop VR learning programs for H&P skills including essential knowledge and competence according to the guidelines of the American Board of Otolaryngology (Tsue 2014). Worked-out samples (Renkl et al 1998) and self-explanation prompts (Chi et al 1989) were used to reinforce the VR learning. A 10-min 360° video (video encoder: H264/Advanced Video Coding; resolution: 3840 pixels × 2160 pixels; framerate: 30 frames/s) with in-camera stitching, capturing 360° audio, and spherical stabilization using a 360° camera (Garmin VIRB 360, Garmin Ltd., Kansas City, MO, USA) was used to construct the content and scenario of a real-life clinical setting (Chao et al 2021). The first portion of the video demonstrated the skills of history taking in a static situation (Part I), and the second portion demonstrated the skills of conducting a physical examination in a dynamic situation (Part II). Table 1 summarizes the content of the 360° VR learning program. In this study, “static situation” refers to when the camera was held steady as the 360° video was filmed, whereas “dynamic situation” refers to when the camera moved quickly with the educator’s head.

**Table 1:** Summary of the contents of the 360° virtual reality training program

Period	Timestamp	Content	Subject movement
Part I. History taking			
Static	0.0 min–0.5 min	How to protect yourself while facing a real patient during a teaching clinic	Sitting still
	0.6 min–1.0 min	How to obtain previous medical history from the Healthcare/Hospital Information System	Sitting still
	1.1 min–2.5 min	What is the general framework for history taking?	Sitting still
	2.6 min–3.5 min	How to start with the opening greeting and actively listen to the patient	Sitting still
	3.1 min–4.0 min	How to ask the patient with open questions, questions with options, or leading question	Sitting still
	4.1 min–5.0 min	How to summarize and confirm the history of the patient	Sitting still
Part II. Physical examination			
Dynamic	5.1 min–6.0 min	How to use regular instruments to perform a physical examination of the head and neck	Slightly fast-moving
	6.1 min–7.0 min	What are the indications, relevant anatomy, and procedural techniques?	Slightly fast-moving
	7.1 min–7.5 min	How to obtain agreement	Slightly fast-moving
	7.6 min–8.0 min	How to prepare a physical examination of the head and neck	Moderately fast-moving
	8.1 min–8.5 min	How to determine the examination areas	Moderately fast-moving
	8.6 min–9.0 min	How to perform a physical examination safely	Moderately fast-moving
	9.1 min–9.5 min	When to seek help	Slightly fast-moving
	9.6 min–10.0 min	How to explain the examination findings	Slightly fast-moving

Subsequently, the 360° video file was produced using PowerDirector software version 16 (Cyberlink Corp., New Taipei, Taiwan) to adjust the resolution with 3840 pixels × 1920 pixels (video encoder: H264; framerate: 30 frames/s; keyframe frequency: 60 frames; quantization parameter: 4), and courseware was developed to accompany the 360° VR video using Unity 2017.3.1 Editor (Unity Technologies, San Francisco, CA, USA). The 360° video module was developed to arbitrarily review the immersive, 3D, and 360° VR video through an HMD (Vive VR headset, HTC Corp., New Taipei, Taiwan).

The students were immersed in the 360° experience to learn the H&P skills. Before the 360° VR learning, the functionality of the VR device was explained to the participants. The students were able to review the instructor’s demonstrations and responses of standard patients and other medical staff from a first-person perspective in an immersive 360° environment at any time and for as many times as they wished.

### Measures

#### Autonomic function

Autonomic function was assessed using HRV monitoring. After sitting quietly for 20 min, electrocardiogram signals of a single lead were recorded for 5 min using a Holter-like Nexus-4 amplifier and recording system (MindMedia BV., Herten, The Netherlands) with the participants wearing an HMD and breathing normally to collect baseline data. The electrocardiogram signals were continuously recorded during the 360° learning and 5 min after the end of the learning in a sitting position. Therefore, the levels of physical activity and stress of the participants were controlled.

Signals were recorded at a sample rate of 1024 Hz, and the raw data were saved in CSV format. HRV analysis was performed using a self-developed MATLAB (The MathWorks, Inc., Natick, MA) program that allowed us to determine HRV parameters from sequences of consecutive 5-min epochs of electrocardiogram signals. The HRV analysis followed the requirements of the quality of HRV analysis established by the European Society of Cardiology and the North American Society of Pacing and Electrophysiology (Task Force of the European Society of Cardiology and the North American Society of Pacing and Electrophysiology 1996). For the analysis, sequences of the R wave-to-R wave (RR) intervals were selected without artifacts, ventricular excitations, and supraventricular excitations (Shaffer and Ginsberg 2017).

The power spectrum was quantified by a fast Fourier transform, in which HRV variables are categorized to frequency-domain measurements, including VLF (power between 0.003 and 0.04 Hz), LF (power between 0.04 and 0.15 Hz), high frequency (HF) (power between 0.15 and 0.4 Hz), and total power (TP) (power between 0.0 and 0.4 Hz) (Kuo et al 1999; Task Force of the European Society of Cardiology and the North American Society of Pacing and Electrophysiology 1996). Emotional tension can induce SS and subsequently increase heart rate during simulator training (Min et al 2004). Heart rate can be an indicator of SS, because the heart rate of participants with SS has been shown to be more rapid than those without SS (Cobb et al 1999). VLF power may involve a parasympathetic component (Taylor et al 1998) and is modulated by stress responses during significant emotional stress (McCraty et al 2009). LF power represents both sympathetic and parasympathetic control of heart rate, HF power represents parasympathetic control, and TP represents a combination of both sympathetic and parasympathetic function (Task Force of the European Society of Cardiology and the North American Society of Pacing and Electrophysiology 1996). Because inter-individual variability is large, the ratio of LF to HF (LF/HF) is frequently used to quantify sympathovagal balance (Burr 2007).

#### Mental workload

After completing the 360° VR learning program, each participant provided a self-reported mental workload measure using the NASA TLX questionnaire (Hart and Staveland 1988). The VR learning program was performed in a manner somewhat similar to a personal computer video game. Therefore, the NASA TLX questionnaire was administered to assess an individual’s mental workload because of its good reliability and validity in computer operation tasks (Yang and Deng 2010). This instrument consists of six subscales: mental demand, physical demand, temporal demand, performance, effort, and frustration. Participants rate the level of each dimension by making a mark on a visual analogue scale (range, 0–20).

#### Assessment of history taking and physical examination skills

After completing the NASA TLX questionnaire, each participant performed a real-patient H&P for a maximum of 20 min at a teaching clinic and was evaluated with a mini-CEX by an investigator blinded to the information on SS. Mini-CEX is a feasible and reliable tool to evaluate the H&P skills of residents and undergraduate medical students in realistic clinical encounters (Hauer 2000; Durning et al 2002; S. Kim et al 2016), in which an instructor directly observes a learner performing a focused clinical skill and do the assessment simultaneously. A “real patient” was defined as a patient with an actual but relatively stable medical condition, so that the effects of patient diversity on the Mini-CEX results would be minimized (S. Kim et al 2016). The participant’s competency of H&P was assessed with a Mini-CEX rating form (range: 7–63) (Chang et al 2013; Norcini 1995), which contained seven clinical competencies: (1) medical interviewing; (2) physical examination; (3) professionalism; (4) clinical judgment; (5) counseling skills; (6) organization/efficiency; and (7) overall clinical competence. The results were also assessed for the satisfaction of both the teacher and student using a nine-point rating scale (1 = unsatisfactory and 9 = superior) (Chang et al 2013; Kogan et al 2002). Reflections were encouraged at the end of the Mini-CEX to ensure learning effectiveness (Mawdesley et al 2011).

#### Measurement of simulator sickness

After completing the VR learning program, each participant in this study immediately provided qualitative feedback about their experience of the 360° VR application regarding 16 SS symptoms before real-patient H&P and Mini-CEX. Without any of the 16 SS symptoms, the participants were categorized into the SS (-) group. In cases that had any SS symptom, they were further immediately and orally evaluated according to the SSQ by research team members. The SSQ is a validated method of assessing SS (Kennedy et al 1993) and is considered to be the gold standard assessment tool for SS symptoms (Keshavarz and Hecht 2011a, 2011b). The 16 SS symptoms were general discomfort, fatigue, headache, eye strain, difficulty focusing, increased salivation, sweating, nausea, difficulty concentrating, fullness of head, blurred vision, dizzy (eyes open), dizzy (eyes closed), vertigo, stomach awareness, and burping. Each symptom score was rated as 0 (none), 1 (mild), 2 (moderate), or 3 (severe), and the total SSQ score was calculated by adding the 16 symptom scores and multiplying the sum by 3.74 (Kennedy et al 1993). Traditionally, a total score greater than 20 has been shown to represent “significant SS” and a total score less or equal to 20 but more than 0 has been shown to represent “significant SS” (Treleaven et al 2015; Webb et al 2009). In this study, the participants were further categorized into the “SS (−)” subgroup (i.e., total SSQ score = 0) or the “SS (+)” subgroup (i.e., total SSQ score ≥ 3.74).

According to the participants’ feedback, they reflected that the fast movement of the video content made them perceived SS. To explore the temporal associations among fast movement, SS, and HRV, the participants were asked about the onset and progression of their most representative SS symptom after they had re-reviewed the 360° VR video. Subsequently, the severities of SS (the most representative symptom scores) at the 0, 2nd, 4th, 6th, 8th, and 10th min were recorded.

Meanwhile, the degree of fast movement at different timestamps was measured on a three-point scale (0: sitting still; 1: slightly fast-moving; 2: moderately fast-moving) by two of the authors (Table 1).

### Main outcome measurements

The primary outcome measurement of this study was the total SSQ score after the 360° VR H&P learning. Other outcomes including HRV indices, NASA TLX subscales, and Mini-CEX scores were also collected.

### Data analysis

The D'Agostino and Pearson normality test showed that most of the continuous variables were non-normally distributed. Therefore, the descriptive statistics of normally distributed variables were presented as means and standard deviations, and skewed variables were presented as median and interquartile range (IQR). For normally distributed variables, differences between the SS (+) and SS (−) subgroups were analyzed using the independent-samples *t* test and differences within the SS (+) or SS (−) subgroups the paired-samples *t* test. For skewed variables, the Mann–Whitney *U* test was used for comparisons between two subgroups, and the Wilcoxon signed rank test for comparisons of related samples. For comparing with a reference value of “10” of NASA TLX subscales, one-sample *t* test or one-sample nonparametric test was applied, as appropriate. Differences in categorical variables between two subgroups were analyzed using Fisher’s exact test. Associations between normally distributed variables and skewed variables were analyzed using the Spearman correlation test, and the relationships of categorical variables with normally distributed or skewed variables were analyzed using the Point-Biserial correlation test. *P*-values were two-sided, and statistical significance was accepted at *p* < .01 to reduce the overestimation of multiple comparisons.

A multilevel model test was used to determine the likelihood that a number of variables had an effect on the relationship between total SSQ score and interesting variables of nested structure data. Herein, a two-sided *p-*value .05 was considered statistically significant. All statistical analyses were performed using GraphPad Prism 9.0 for Windows (GraphPad Software Inc., San Diego, CA, USA) and SPSS 25.0 statistical package for Windows (International Business Machines Corp., Armonk, NY, USA).

## Results

### Participants’ characteristics

None (0%) of the participants dropped out from the 360° VR learning program. Eight (29%) females and 20 (71%) males with a median age of 24 (IQR, 24–25) years were included for statistical analysis.

### Simulator sickness

Ten (36%) students had at least one SS symptom during the 360° VR learning, and 18 (64%) students had no SS throughout the learning. Herein, we categorized the participants who had at least one SS symptom into the SS (+) subgroup and those without any SS symptom into the SS (-) subgroup. There were no significant differences in age (24 [24–24] years vs 24 [23–25] years, *p* = .76) and sex (female/male: 3/7 vs 5/13, *p* > .99) between the SS (+) and SS (−) subgroups at baseline.

**Fig. 2:**
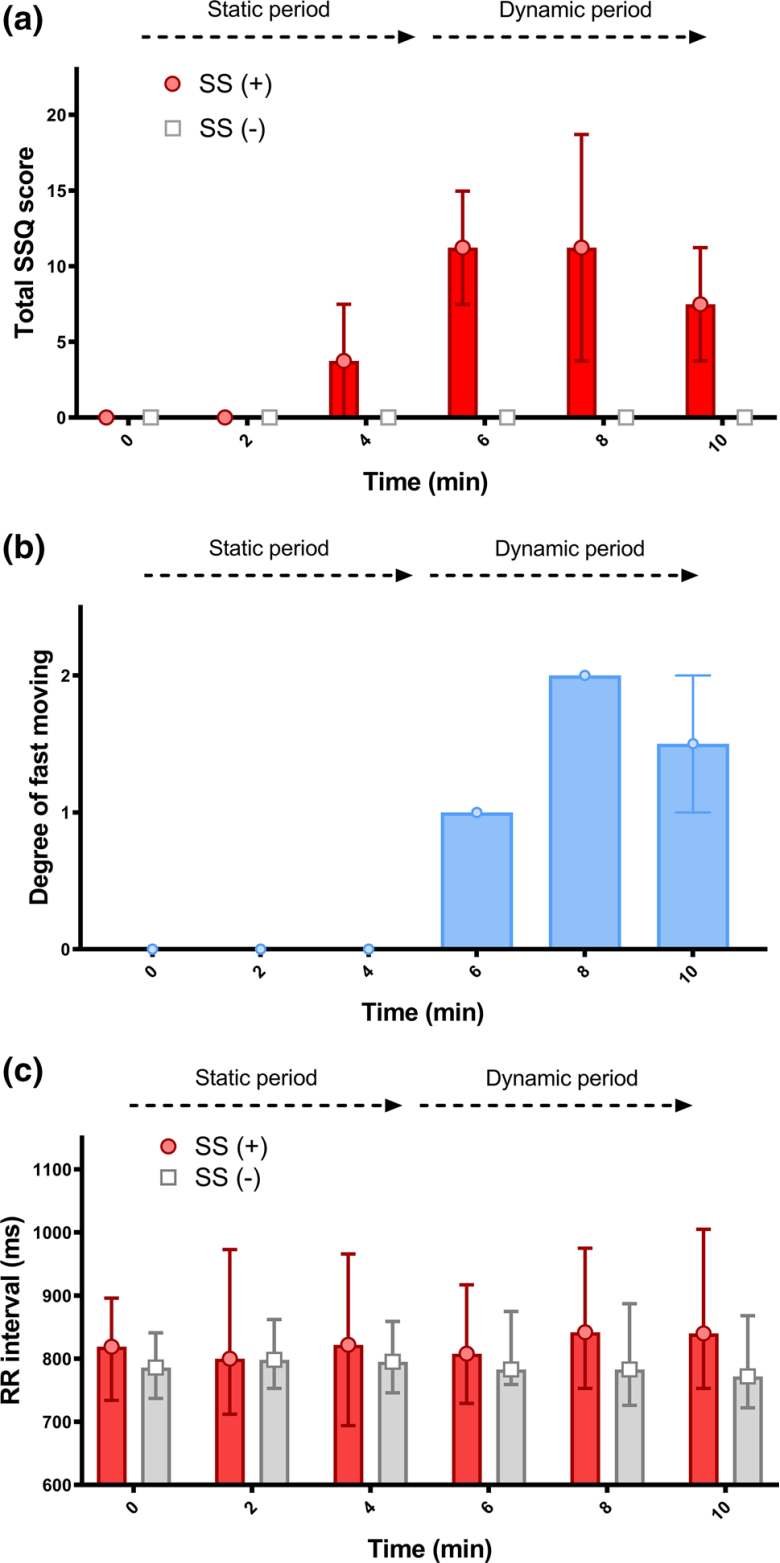
Distributions of (**a**, upper) total Simulator Sickness Questionnaire (SSQ) scores between the simulator sickness (SS) (+) and SS (−) subgroups, (**b**, middle) the degree of fast moving of the 360° virtual reality video, and (**c**, lower) R wave-to-R wave (RR) intervals between the SS (+) and SS (−) subgroups during 360° virtual reality learning. Data are summarized as medians (upper and lower limits)

In the SS (+) subgroup, six (60%) had dizziness (eyes open), five (50%) had difficulty focusing, five (50%) had difficulty concentrating, and one (10%) had vertigo. Therefore, five (45%) had nausea-related subscore, six (55%) had oculomotor-related subscore, and seven (64%) had disorientation-related subscore. The median total SSQ score was 14.96 (IQR: 7.48–14.96). The students with SS reported that the SS began in the 4th min before the dynamic video clips, and then reached maximum severity in the 8th min (Fig. 2a).

The degree of fast moving began in the 6th min and reached the highest degree in 8^th^ min (Fig. 2b). There was a significant association between the total degrees of fast moving and the total SSQ score (*r* = 0.32, *p* < .001).

### Autonomic function

Table 2 shows the HRV indices for frequency domain that were measured at baseline, 0–5 min, and 6–10 min. VLF power-6–10 min and TP-6–10 min in the SS (+) subgroup were significantly larger than those in the SS (−) subgroup (*p* = .002 and .009, respectively).

**Table 2:** Summary of heart rate variability parameters

Characteristics	Overall	Simulator sickness (+)	Simulator sickness (-)	*P*-value^a^
N	28	10	18	
RR interval-baseline, ms	799 ± 96	824 ± 108	784 ± 89	.310
RR interval-0–5 min, ms	806 ± 108	822 ± 137	797 ± 92	.570
RR interval-6–10 min, ms	802 ± 106	827 ± 120	787 ± 99	.352
VLF power-baseline, ms^2^	1026 (586–2982)	2879 (902–4068)	902 (463–1377)	.040
VLF power-0–5 min, ms^2^	1165 ± 644^c^	1526 ± 544^c^	964 ± 618	.024
VLF power-6–10 min, ms^2^	1356 (799–2027)^c^	2050 (1551–1945)^c^	978 (656–1517)	.002
LF power-baseline, ms^2^	1609 (744–2164)^b^	1938 (1598–3845)^b^	1315 (673–1945)	.040
LF power-0–5 min, ms^2^	908 (663–1451)^b^	1250 (623–1550)^b^	856 (676–1325)	.524
LF power-6–10 min, ms^2^	1060 (759–1353)^b^	1325 (779–2424)	991 (731–1284)	.133
HF power-baseline, ms^2^	812 (536–1141)	924 (490–1212)	798 (540–1127)	.759
HF power-0–5 min, ms^2^	741 (418–1009)	821 (625–1199)	637 (367–927)	.308
HF power-6–10 min, ms^2^	806 (385–1175)	927 (738–1618)	524 (316–1023)	.133
TP power-baseline, ms^2^	3491 (2547–6897)^b^	6888 (3283–6993)	3216 (2316–4205)	.040
TP power-0–5 min, ms^2^	3162 (1902–4128)^b^	3163 (3150–4465)^c^	2997 (1583–3997)	.245
TP power-6–10 min, ms^2^	3607 (2418–3798)	3750 (3725–6855)^c^	2914 (2035–3746)	.009
LF/HF ratio-baseline	2.36 ± 1.58^b^	2.95 ± 1.62	2.03 ± 1.49	.141
LF/HF ratio-0–5 min	1.56 (0.72–2.50)^b^	1.19 (0.81–2.58)	1.68 (0.62–2.36)	> .999
LF/HF ratio-6–10 min	1.84 (1.00–2.84)	1.55 (0.96–2.31)	1.85 (0.99–3.77)	.724

Data are summarized as means ± standard deviations or medians and interquartile ranges or numbers (percent), as appropriate

Abbreviations: HF: high frequency; LF: low frequency; LF/HF: low frequency/high frequency; RR: R wave-to-R wave interval; TP: total power; VLF: very low frequency power

^a^Data were compared using the independent *t* test or Mann–Whitney *U* test (two-tailed), as appropriate

^b^*P* < .01, compared with a baseline value, the paired-samples *t* test or Wilcoxon signed-rank test (two-tailed)

^c^*P* < .01, compared with a value of 0–5 min, the paired-samples *t* test or Wilcoxon signed-rank test (two-tailed)

In the overall group, changes in the RR interval and HF power were not statistically significant across different time points. VLF power-6–10 min was significantly larger than VLF power-0–5 min (*p* < .001). Both LF power-0–5 min and LF power-6–10 min were significantly smaller than LF power-baseline (*p* = .002 and .009, respectively). TP-0–5 min power and LF/HF ratio-0–5 min were significantly smaller than TP-baseline power and LF/HF ratio-baseline (*p* = .007 and .009, respectively).

Changes in the RR interval (Fig. 2c) were not statistically significant across different time points and subgroups. In the SS (+) subgroup, VLF power-6–10 min power and TP-6–10 min were significantly larger than VLF power-0–5 min and TP-0–5 min (*p* = .005 and .007, respectively), and LF power-0–5 min was significantly smaller than LF power-baseline (*p* = .005). Furthermore, there was no statistically significant difference in HRV indices across different time points in the SS (-) subgroup.

### Mental workload

Subscales of the NASA TLX in the overall group were comparable with a reference of “10” (see Table 3). Comparing with the reference values, the SS ( +) subgroup had significantly larger physical demand score (*p* = .007), whereas the SS (−) subgroup had a significantly smaller frustration score (*p* = .002). Furthermore, the physical demand and frustration scores of the SS (+) subgroup were significantly larger than those of the SS (−) subgroup (*p* = .005 and .001, respectively).

**Table 3:** Summary of the NASA Task Load Index subscales

Characteristics	Overall	Simulator sickness ( +)	Simulator sickness (-)	*P*-value^a^
N	28	10	18	
Mental demand	13 (10–15)^b^	13 (11–15)	13 (7–15)	.654
Physical demand	10.6 ± 4.7	13.8 ± 3.0^b^	8.8 ± 4.6	.005
Temporal demand	10 (8–12)	10 (9–12)	10 (8–12)	.832
Performance	10 (5–15)	12 (6–14)	10 (5–15)	.689
Effort	13 (10–14)	12 (11–14)	13 (10–14)	.689
Frustration	8.0 ± 5.6	12.3 ± 4.9	5.7 ± 4.4^c^	.001

Data are summarized as means ± standard deviations or medians and interquartile ranges, as appropriate.

^a^Data were compared using the independent t-test or Mann-Whitney U test, as appropriate.

^b^*P* < .01compared with a reference value (“10”), one-sample t-test or one-sample nonparametric test (two-tailed), as appropriate.

### Skills of history taking and physical examination

The physical examination and counseling skills in the SS (+) subgroup were significantly smaller than those in the SS (−) subgroup (*p* = .004 and .006, respectively). There were no significant differences in other item scores of the Mini-CEX between the two subgroups (see Table 4).

**Table 4:** Summary of the Mini-Clinical Evaluation Exercise variables

Characteristics	Overall	Simulator sickness (+)	Simulator sickness (-)	Unadjusted *p*-value^a^
N	28	10	18	
Medical interview	6 (5–6)	5 (5–7)	6 (5–6)	.569
Physical examination	5 (5–6)	5 (4–5)	6 (5–6)	.001
Professionalism	6 (5–7)	6 (6–7)	6 (5–7)	.518
Clinical judgment	6 (5–6)	6 (5–6)	6 (5–6)	.888
Counseling skills	6 (5–6)	5 (5–6)	5 (6–7)	.002
Organization/efficiency	6 (5–6)	6 (5–7)	6 (5–6)	.677
Overall clinical competence	6 (5–6)	5 (5–6)	6 (5–6)	.013
Teacher’s satisfaction	9 (8–9)	8 (8–9)	9 (9–9)	.018
Learner’s satisfaction	9 (9–9)	9 (9–9)	9 (9–9)	.426

Data are summarized as medians and interquartile ranges

^a^Data were compared using the Mann–Whitney *U* test

### Associations among total simulator sickness questionnaire score with interested variables

**Fig. 3:**
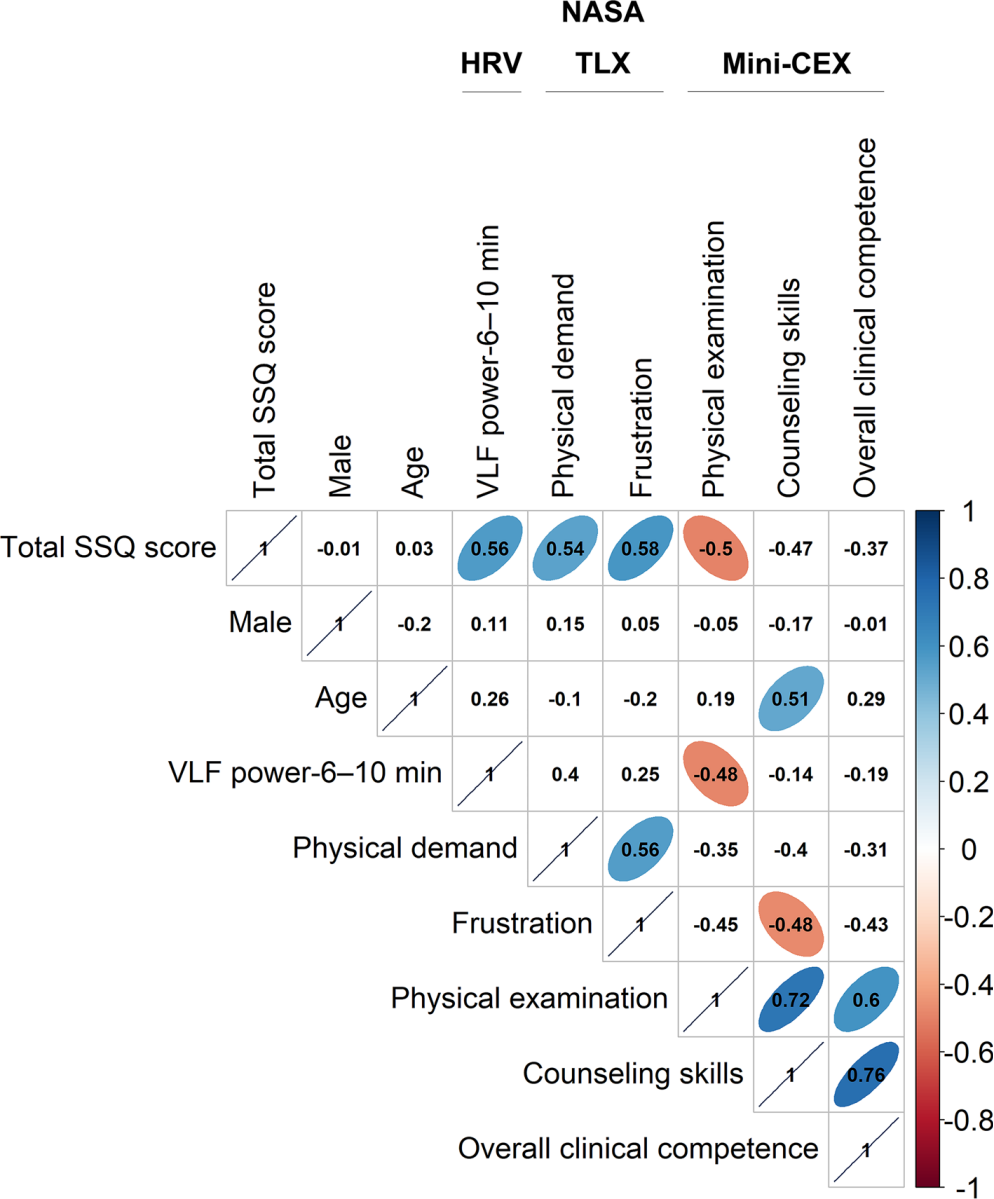
Significant associations among total Simulator Sickness Questionnaire (SSQ) score, heart rate variability (HRV) indices, NASA Task Load Index (NASA TLX) subscales, and Mini-Clinical Evaluation Exercise (Mini-CEX) scores in a 360° virtual reality application for learning history taking and physical examination skills (two-sided *p*-values < .01). Spearman correlation coefficient values between normally distributed variables and skewed variables or Point-Biserial correlation coefficient values among categorical, normally distributed, and skewed variables are displayed in the boxes as appropriate. Abbreviations: TP: total power; VLF: very low frequency

Significant correlations were found among total SSQ score and several variables (Fig. 3).

First, total SSQ score was positively associated with VLF power-6–10 min (*r* = 0.56, *p* = .002), supporting Hypothesis 1. In addition, VLF power-6–10 min was significantly inversely correlated with physical examination score (*r* =  − 0.48, *p* = .0095).

Second, total SSQ score was positively related to physical demand subscale (*r* = 0.54, *p* = .006) and frustration subscale (*r* = 0.58, *p* = .001), supporting Hypothesis 2. In addition, frustration subscale was inversely correlated with counseling skills score (*r* =  − 0.48, *p* = .0098).

Third, total SSQ score was inversely related to physical examination score (*r* = -0.50, *p* = .002), supporting Hypothesis 3. Moreover, age was associated with counseling skills score (*r* = 0.51, *p* = .005).

### Multilevel model test

**Fig. 4:**
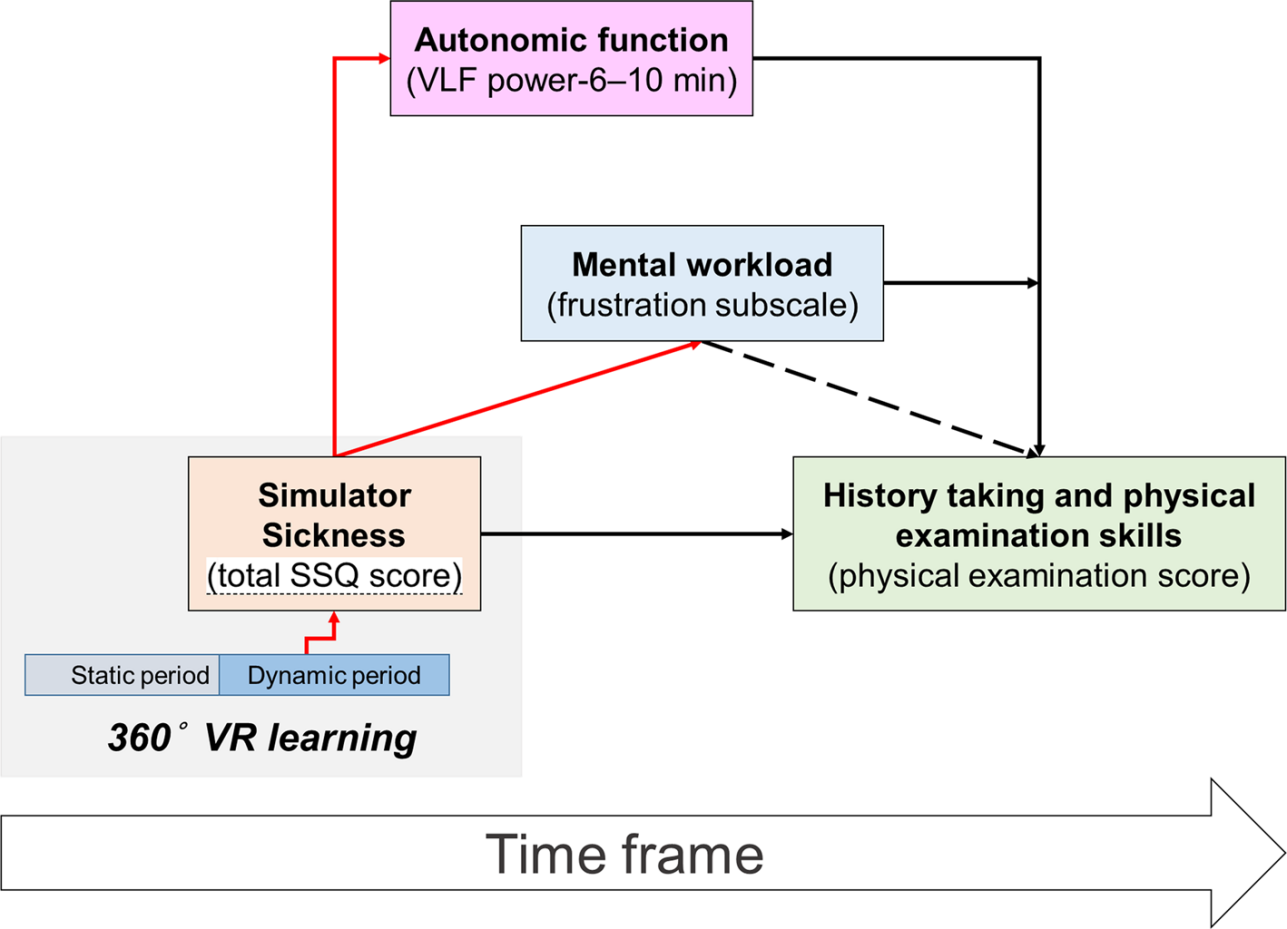
Multilevel moderated mediation models of the relationships among simulator sickness (in terms of total Simulator Sickness Questionnaire [SSQ]), history tacking and physical examination skills (in terms of physical examination score), autonomic function (in terms of very low frequency [VLF] power), and mental workload (in terms of frustration subscale). Red/black consolidation lines indicate positive/inversive relationships with two-sided *p*-values < .05, whereas a dashed line indicates a relationship with a two-sided *p*-value ≥ .05

Briefly, total SSQ score was inversely related to physical examination score, VLF power-6–10 min was inversely associated with physical examination score, and total SSQ score was positively related to VLF power-6–10 min (Fig. 4). Therefore, VLF power-6–10 min could mediate the relationship between total SSQ score and physical examination score. Accordingly, the multilevel model test was performed for the nested data structure. The raw total SSQ score, VLF power-6–10 min, fluctuation subscale, and physical examination score had been normalized. Using the linear mixed models, we estimated the maximum likelihood. We computed that the intra-class correlation coefficient (1,1) (LeBreton et al. 2008) was 0.278 (0.285 / [0.285 + 0.742]). The likelihood ratio was 4.678 (80.171–75.493), whereas the 5% point of a Chi-squared distribution on 1 degree of freedom was 3.84. Therefore, we further performed multilevel modeling with SS effects. We included VLF power-6–10 min to extend the total SSQ score effects model. Of random intercept models, the VLF power-6–10 min estimate (standard errors [SE]) was −0.370 (0.179) (*p* = .048). The intra-class correlation coefficient (1,1) was 0.138 (0.112 / [0.112 + 0.702]). Using random slope models, the VLF power-6–10 min estimate (SE) was −0.428 (0.176) (*p* = .038). Although frustration subscale did not significantly extend the total SSQ score effects model (*p* > .05), VLF power-6–10 min (estimate,  − 0.404; SE, 0.163; *p* = .020) and frustration subscale (estimate, −0.066; SE, 0.292; *p* = .032) did using random intercept models.

## Discussion

In this study, we examined differences in HRV indices, NASA TLX subscales, and Mini-CEX scores between medical students who did and did not have SS during 360° VR learning. Based on the principles of medical education and previous VR research, we hypothesized that we would observe significant differences between the two groups of students. Significant changes in LF power before SS and VLF power and TP during SS indicated that the students with SS might have had different ANS characteristics. Notably, larger total SSQ score was significantly related to larger VLF power-6–10 min, larger physical demand subscale and frustration subscale, and smaller physical examination score. Our findings supported our three hypotheses. In addition to that learners’ SSQ score negatively predicted their physical examination performance after a 360° VR H&P learning, VLF power-6–10 min mediated the relationship between total SSQ score and physical examination score. Total SSQ score was positively related to frustration subscale, and the latter one moderated the mediating effect of VLF power-6–10 min in the relation between total SSQ score and physical examination score (Fig. 4). Therefore, documenting SS is essential to evaluate the quality of VR applications for medical education.

Accordingly, understanding the causes of SS during a 360° learning program is important for improving the quality of this innovative educational tool. Because SS is a similar syndrome to motion sickness, Brooks (Brooks et al 2010) and Dużmańska (Duzmanska et al 2018) extended several theories of motion sickness, including the Evolutionary Theory, Sensory Conflict Theory, and Postural Instability Theory, to explain SS. The Evolutionary Theory indicates that the users have less time to adapt to the VR condition (Treisman 1977). The Sensory Conflict Theory considers that the sensory information received does not match one’s previous experiences (Reason and Brand 1975). The Postural Instability Theory suggests that the users cannot adjust themselves to postural instability to maintain a sense of balance (Riccio and Stoffregen 1991). Therefore, frequent usages of VR applications for medical education can help the students to increase exposure to the VR environment, enhance the VR learning experience, and improve postural instability to adapt to the VR condition.

As the negative impact of SS on mental workload and learning outcomes has been largely addressed in the literature, the associations of SS-related HRV indices with mental workload and learning outcomes would be more interesting. Our findings related to HRV can be summarized as follows: (1) The students could have the largest LF power after wearing the HMD at baseline and soon reduced, particularly in SS-positive ones (Table 2). (2) The dynamic 360° VR video could activate the ANS to increase VLF power and TP during the 360° VR learning, especially in SS-positive students. (3) The total SSQ score was positively associated with large VLF power-6–10 min. (4) Interestingly, VLF-6–10 min was inversely associated with physical examination score.

This could be because the undergraduate medical students were able to overcome the challenge of the VR condition and the SS symptoms via regulating ANS control (mainly the parasympathetic nervous system) to quickly recover during and after the 360° VR learning. This hypothesis is further discussed in the following paragraphs.

Although the HRV indices-baseline were comparable between the SS (+) and SS (−) subgroups, the participants with a significant reduction in the LF power in the static period were more likely to have SS during the 360° VR learning. VLF remained unchanged after exposure to the static 360° VR video but significantly increased after reviewing the dynamic 360° VR video in the SS (+) subgroup. Notably, the students with SS had larger VLF power during the 360° VR learning and VLF power has been reported to be an indicator of delayed recovery after mental stress (Usui and Nishida 2017). These findings indicated that an unstable autonomic control may predispose the occurrence of SS. In addition, the participants with SS may have higher TP by activating the sympathetic nervous system as well as parasympathetic activity (Task Force of the European Society of Cardiology and the North American Society of Pacing and Electrophysiology 1996). Our findings also suggest that SS may affect the ANS in a manner similar to motion sickness. Overall sympathetic activation with increasing motion sickness could produce a significant increase in vagal activity before the most severe level of motion sickness (Lacount et al 2009).

The literature review was inconclusive with regard to the physiological importance of VLF power. It may represent an index of thermogenic sympathetic nervous activity (Matsumoto et al 2001) or depend on the presence of parasympathetic outflow (Taylor et al 1998). A 3D video has been reported to induce larger heart rate and VLF/HF ratio than a 2D video through the activation of sympathetic activity (S. Park et al 2014). These findings might also suggest that the participants needed to produce a higher parasympathetic function to overcome the mild SS during 360° VR learning.

We observed that these participants frequently performed self-adjustments such as deep breathing, swallowing, and retching (Lin et al 2013) to effectively reduce the sympathetic activities while reviewing the static 360° VR video. However, they watched the dynamic 360° VR video and experienced SS despite continuously performing self-adjustments. Although some users might recover from SS by adapting to repetitive or prolonged VR exposure, most cases have been reported to experience more severe syndrome after longer exposure (Duzmanska et al 2018). Therefore, participants with SS might need other methods to improve ANS imbalance after wearing an HMD and before 360° VR learning. For example, both mind–body exercise and transcutaneous electrical nerve stimulation can reduce stress, SS, and LF/HF ratio (Chu et al., 2018).

In this study, we did not perform ultra-short HRV analysis. Short-term HRV analysis has been widely investigated in several studies, whereas the validity of ultra-short HRV features remains unclear (Castaldo et al 2019). Currently, 5-min recordings are regarded as being appropriate for HRV analysis to detect SS and mental stress in healthy subjects. In a review paper (Pecchia et al 2018), the authors stated that rigorous methods are lacking to assess the validity of ultra-short HRV features in a control situation and to identify reliable ultra-short HRV features. Moreover, we aimed to identify relationships between HRV indices and the progression of SS during 360° VR learning in this study, and real-time HRV processing was not our purpose. Based on these reasons, we only used short-term HRV analysis in this study.

As peripheral viewing of a poorly depicted background element of 3D video clips could exacerbate SS symptoms (Takada et al 2015), our pre-pilot workups found a low resolution of 360° VR video (1920 pixels × 960 pixels) could induce moderate-to-severe SS in our volunteers. To reduce the intensity of SS, we need to design a high-quality 360° VR application, including high-resolution 360° videos, high-end HMDs, and ergonomic VR software, to provide deeper immersion, better quality graphics and sound, and more helpful instructions and prompts (Kourtesis et al 2019). High-end HMDs should enhance their accommodation and dynamic depth-of-field (Carnegie and Rhee 2015), and reduce overheating and blue light (M. J. Park et al 2019). However, these approaches can simultaneously increase presence and SS (Weech et al 2019). Furthermore, dynamic situations could lead to more severe SS in this study, and therefore the movement of the camera with the educator’s head should be reduced. In addition, the maximum duration of VR sessions should also be limited to between 55 and 70 min to avoid the occurrence of SS (Kourtesis et al 2019). These hardware and software advances will allow the students to learn more quickly, interpret situations more accurately, and accomplish VR learnings without delay (Silva et al 2018).

The main contributions of this study include: (1) improving the validity of a three-point HRV-based physiological measurement of SS in a 360° VR application for medical education, (2) discovering differences in a wide range of HRV indices, mental workload subscales, and training outcomes between SS subgroups, (3) elucidating potential mechanisms of the ANS and SS, and (4) developing several practical strategies based on HRV measurement to diminish the occurrence and severity of SS for 360° VR training programs.

### Limitations

There were several limitations to the study. First, it was a post hoc retrospective investigation using a between-subject design. In our original randomized controlled trial (ClinicalTrials.gov Identifier: NCT03501641), mild but significant SS during the 360° VR training program was reported by the participants, after which we consequently collected qualitative feedback about their onset and progression of SS. A recall bias might have existed. A think-aloud protocol using the controllers during the VR experience to obtain real-time measures of SS will be more suitable in usability testing in the design and development of VR training applications (Koch et al. 2019). Second, a selection bias may have existed concerning the single Han ethnicity of the subjects, a predominance of males in our study population, and a subgroup of healthy students. Third, the sample size was not very big, and the age spectrum was limited to 23–26 years. Lastly, there was no control group, which may have limited the generalizability of the study results. The research team made the best effort to report all the relevant results based on the understanding that a demonstration of only positive findings would lead to publication bias. This study revealed that 36% (10/28) of the participants had SS, which was clinically significant and presented a considerable portion of the sample. Our findings yielded meaningful instructional implications of that SS was not uncommon and should be weighed with other advantages and disadvantages when applying VR in medical education on H&P skills.

Although we used a relatively strict *p*-value and multilevel models to reduce the type I and type II errors in such a nested data structure, future studies should include a larger sample size to properly estimate the models. Also, despite none of our participants had diagnosed mental or psychiatric conditions, stress level (H. G. Kim et al 2018a, b), anxiety disorders (Chalmers et al 2014), or concussion (Blake et al 2016) that could have possibly impacted the HRV indices and SS (Duzmanska et al 2018) can be included in future studies to further control possible confounding factors. Future research should investigate these practical strategies based on HRV measurements to diminish the occurrence and severity of SS for 360° VR training programs.

## Conclusions

During the COVID-2019 pandemic, VR applications for medical education may help medical students to learn outside the hospital. Notably, total SSQ score was positively associated with VLF power-6–10 min, physical demand subscale, and frustration subscale, and inversely related to physical examination score in the 360° VR application for medical education. Therefore, identification and management of SS are important for evaluating and improving the quality of 360° VR training programs. In addition, VLF power has a considerable potential to objectively monitor the occurrence and progression of SS than other subjective SS measurements in healthy young adults, especially during VR applications. The combination of HRV analysis with the mental workload and SS measurements and outcome assessments provided the best clinical value to evaluate the VR applications for medical education in this study.

## Data Availability

The datasets generated during and/or analysed during the current study are available from the corresponding author on reasonable request.
